# Comparison of three different reduction methods of the ankle mortise in unstable syndesmotic injuries

**DOI:** 10.1038/s41598-019-51988-y

**Published:** 2019-10-28

**Authors:** Sven Yves Vetter, Nils Beisemann, Holger Keil, Marc Schnetzke, Benedict Swartman, Jochen Franke, Paul Alfred Grützner, Maxim Privalov

**Affiliations:** BG Trauma Center Ludwigshafen at Heidelberg University Hospital, Ludwigshafen, Germany

**Keywords:** Reconstruction, Experimental models of disease

## Abstract

In order to achieve a clinically satisfying result and to prevent posttraumatic osteoarthritis in the treatment of unstable syndesmotic injuries, anatomically correct reduction is crucial. The objective of the study was to investigate three different reduction methods of the ankle mortise in unstable syndesmotic injuries. In a specimen model with 38 uninjured fresh-frozen lower legs, a complete syndesmotic dissection was performed. The ankle mortise was reduced with either a collinear reduction clamp, a conventional reduction forceps or manually with crossing K-wires. The reduction clamps and the K-wires were placed in a 0°-angle to the leg axis. The clamps were positioned on the posterolateral ridge of the fibula 20 mm proximal to the ankle joint line. A cone beam computed tomography was performed after dissection and after each reduction. Tibio-fibular distances and angles were determined. Despite significant differences in terms of overcompression (0.09–0.33 mm; p = 0.000–0.063) and the slight external rotation (0.29–0.47°; p = 0.014–0.07), the results show a satisfying reduction of the ankle mortise. There were no considerable differences between the reduction methods. It can therefore be concluded that the ankle mortise can be reduced with any of the methods used, but that the positioning and the contact pressure must be considered.

## Introduction

The incidence of ankle fractures is about 180 fractures per 100,000 people per year^1,2^. The number, especially among older people (=/>60 years), is comparatively increasing^3^. Nearly every seventh ankle fracture is accompanied by a syndesmotic injury^4^. In athletes, it was observed that in severe ankle sprains with ligamentous injuries, 20% presented with syndesmotic injuries^5^. The age peak for isolated syndesmotic injuries ranges between 18 and 34 years. The young (0–17 years) are more likely to sustain a growth plate injury, while the elderly (>65 years) tend to suffer a fracture^6^. The anatomic congruence of the distal fibula and tibia plays a pivotal role in the stability of the ankle joint and the force transmission to the talus. A change of length, rotation or a lateral shift of the fibula leads to increased contact pressures of the articular surfaces^7,8^. It is well established that anatomic reduction of the ankle mortise in unstable syndesmotic injuries prevents premature osteoarthritis^9–12^. Therefore, the achievement of an anatomic reduction and a stable transfixation of the ankle mortise are the primary objectives of the operative treatment. The diagnosis and therapy of unstable syndesmotic injuries can be challenging for the orthopedic surgeon^13–16^. Especially the anatomic reduction and correct fixation of the ankle mortise can be more complicated than it appears. Studies have revealed up to 52% malreduced ankle joints in postoperative computed tomography (CT)^10,17^. Even with open reduction, a rate of malreduction of 16% was reported^18^. Conventional fluoroscopy with the three standard projections anteroposterior (AP), lateral, and mortise is the gold standard intraoperatively. In these standard views, the width of the joint space, the tibiofibular overlap, and the fibula anterior-posterior translation are analyzed. The limitations of conventional fluoroscopy are disputed, but there is evidence that conventional fluoroscopy is restricted in detecting malreduction of the ankle joint^19,20^. Intraoperative cone beam CT or postoperative CT are strongly recommended to avoid missing ankle malreduction in the treatment of unstable syndesmotic injuries^10,21^.

Besides intraoperative imaging, the reduction techniques of the ankle can present challenges^22,23^. The most common reduction method in the operative treatment of unstable syndesmotic injuries is the conventional bone reduction forceps. Nevertheless, prospective studies have shown that the manual reduction technique is comparable^24^. There are no studies on the collinear reduction clamp for unstable ankle injuries, but good reduction results have been demonstrated in other fracture regions such as the acetabulum^25^. Although the reduction techniques are a fundamental element in the adjustment of the syndesmotic region, transfixation with a positioning screw or a tightrope after anatomically correct reduction is essential^26^. Therefore, minimizing the malreduction rate is crucial.

The objective of the study was to identify the ideal operative reduction method of the ankle joint in a cadaveric model. The hypothesis was that the collinear reduction clamp leads to a superior reduction in the cone beam CT analysis compared to a standard bone reduction forceps or a manual reduction combined with a K-wire transfixation.

## Results

Thirty-eight fresh frozen lower legs of fourteen female and five male cadaver specimens were examined. The average age of the donors was 81.53 years (range: 59–102).

**Figure 1:**
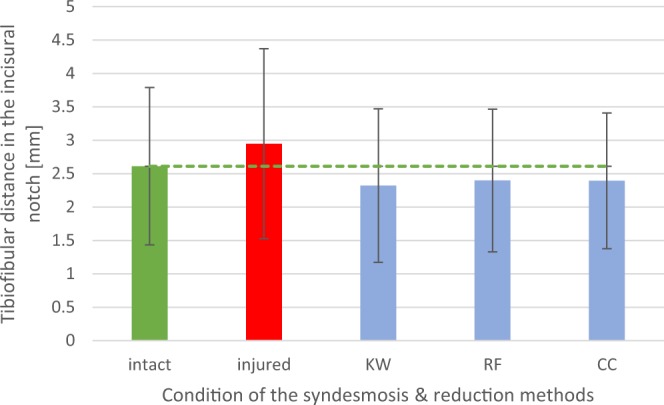
Diagram: Mean tibiofibular distance in the incisural notch (n = 38), depending on the condition of the syndesmotic complex (intact or injured) and the reduction method used (KW = crossing K-wires transfixation; RF = conventional bone reduction forceps; CC = collinear reduction clamp).

**Figure 2:**
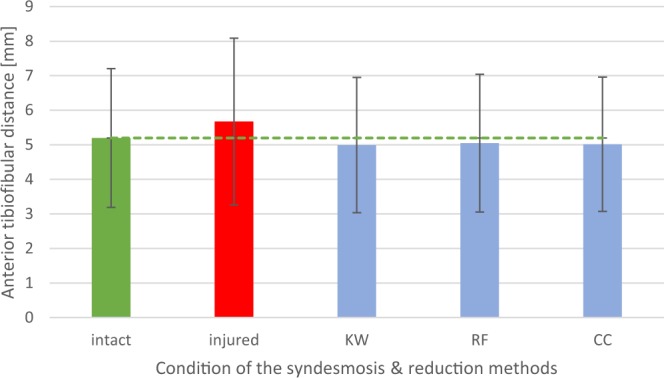
Diagram: Mean anterior tibiofibular distance (n = 38), depending on the condition of the syndesmotic complex (intact or injured) and the reduction method used (KW = crossing K-wires transfixation; RF = conventional bone reduction forceps; CC = collinear reduction clamp).

**Figure 3:**
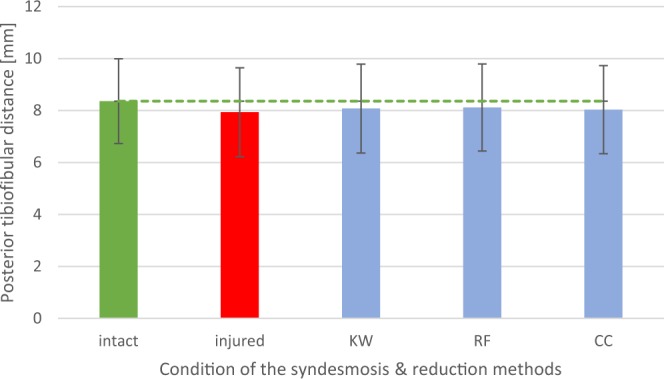
Diagram: Mean posterior tibiofibular distance (n = 38), depending on the condition of the syndesmotic complex (intact or injured) and the reduction method used (KW = crossing K-wires transfixation; RF = conventional bone reduction forceps; CC = collinear reduction clamp).

**Figure 4:**
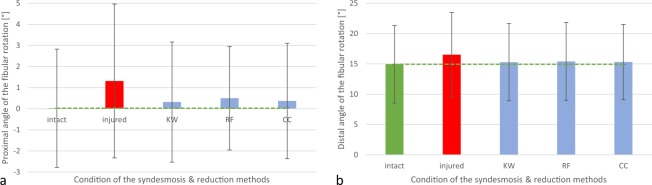
Diagram: Mean (**a**) proximal and (**b**) distal angle of fibular rotation (n = 38), depending on the condition of the syndesmotic complex (intact or injured) and the reduction method used (KW = crossing K-wires transfixation; RF = conventional bone reduction forceps; CC = collinear reduction clamp).

The comparison of the mean values and the standard deviations of the distances and angles in the anatomically intact ankle joint after the dissection of the syndesmotic complex and the reduction using each method are shown in Figs 1–4.

After calculating the average differences of the distances and angles from the intact condition of the ankle joint, the comparison of the reduction methods after the dissection of the syndesmotic complex revealed the following: see Table 1. The reduction methods did not differ considerably from each other. Nonetheless, the collinear reduction clamp demonstrated superior reduction in the TFA (=anterior tibiofibular distance) with a mean remaining difference of 0.09 mm (p = 0.063), while the manual reduction in combination with the K-wire transfixation represented the most appropriate reduction in the PFR (=proximal angle of the fibular rotation) with a mean remaining difference of −0.29° (p = 0.07) and the DFR (=distal angle of the fibular rotation) with a mean remaining difference of −0.34° (p = 0.062). On the other hand, the conventional bone reduction forceps showed the best result when setting the TFI (=tibiofibular distance in the incisural notch) with a mean remaining difference of 0.21 mm (p = 0.003) and the TFP (=posterior tibiofibular distance) with a mean remaining difference of 0.25 mm (p = 0.000).

**Table 1:** T-Test: Mean difference (=MD) and P-values (=p) of the distances (TFI = tibiofibular distance in the incisural notch; TFA = anterior tibiofibular distance; TFP = posterior tibiofibular distance) and angles (PFR = proximal angle of the fibular rotation; DFR = distal angle of the fibular rotation) comparing the intact condition of the syndesmosis and the injured situation after reduction using the three reduction methods (KW = crossing K-wires transfixation; RF = conventional bone reduction forceps; CC = collinear reduction clamp).

Reduction method	TFI		TFA		TFP		PFR		DFR	
	MD in [mm]	p	MD in [mm]	p	MD in [mm]	p	MD in [°]	p	MD in [°]	p
**KW**	0.29	0.001	0.21	0.01	0.29	0.001	−0.29	0.07	−0.34	0.062
**RF**	0.21	0.003	0.15	0.038	0.25	0.000	−0.47	0.027	−0.47	0.037
**CC**	0.22	0.017	0.09	0.063	0.33	0.000	−0.34	0.014	−0.37	0.042

## Discussion

The objective of the study was to identify a superior reduction method of the ankle mortise in unstable syndesmotic injury. The hypothesis posed was that the collinear reduction clamp accomplishes an anatomical reduction of the ankle mortise and is therefore the most suitable reduction method.

The results revealed discrepancies in the reduction methods, but there were no considerable differences. Therefore, the hypothesis of the study has to be refused.

Multiple studies have demonstrated an insufficient reduction of the ankle mortise especially after closed reduction of unstable syndesmotic injuries^17^. With an open reduction, the malreduction rate could be diminished^18,19^. Despite accurate surgical procedures insufficient reduction still could be observed in 6% and 16% of cases with direct visualization of syndesmotic stabilization in ankle fractures respectively^18,19^. Therefore, intraoperative cone beam CT or postoperative CT was recommended to minimize malreduction^10,21^. To technically improve the procedure of anatomic restoration of the ankle mortise we investigated the influence of three different surgical methods on the ankle reduction in a dissected syndesmotic specimen model.

In the literature, the best method for the reduction or transfixation of the syndesmotic region is controversially discussed. Recently, the most common intraoperative reduction technique for injuries of the syndesmotic region is the conventional bone reduction forceps. Nevertheless, prospective studies have shown that the manual reduction technique is comparable^24^.

Fort *et al*.^27^ summarized the relevant anatomy and biomechanics of the ankle joint, the injury mechanism, the diagnosis and treatment of syndesmotic injuries. If instability of the syndesmosis is detected intraoperatively, reduction should be performed primarily with reduction forceps or K-wire, whereby care should be taken not to cause any deterioration of the joint position. The authors recommended a control by intraoperative 3D imaging or fluoroscopy and a transfixation of the syndesmotic region using a positioning screw or the suture button technique, whereby there was no consensus on optimal reduction^27^. This study also supplements the results of the current study, the reduction method did not seem to be the decisive factor, rather it is important not to cause a misalignment during the reduction procedure.

In the cadaveric study by LaMothe *et al*.^28^, comparing the transfixation of syndesmosis using a positioning screw with and without conventional reduction forceps (manually combined with crossing K-wires) and the “suture button technique”, the combination with manual reduction and transient K-wire transfixation showed the best results. However, the authors described the differences as minor, similar to our results, and stated that none of the three reduction methods achieved optimal results^28^. Furthermore, in accordance to our study, a complete correction of syndesmosis without significant differences could not be achieved.

Miller *et al*. noted an overcompression of the ankle mortise and a fibular external rotation of 1.5° in the neutral clamp position. The ankle mortise was less overcompressed with a clamp anterior angulation of 15° or 30° but showed an increased external rotation of the fibula^22^. In consensus with Miller *et al*.^22^, the results of our study indicate that the reduction of the tibiofibular distance leads to minor overcompression, while the fibula in the incisural notch remains slightly rotated outwards. This observation is supported by the results of Phisitkul *et al*. who observed the best reduction of the ankle mortise in a neutral clamp position^29^. On the other hand, the findings of Tornetta *et al*.^12^, who did not detect an overcompression of the ankle mortise after placing a tibiofibular cortical lag screw in a specimen model, could not be confirmed^12^. In this respect, it would be desirable to determine the optimal positioning and contact pressure of the reduction tool in further studies so that the reduction result can be improved in the long term.

Although the reduction methods are a fundamental component in the restoration of the syndesmotic region, transfixation with a positioning screw or a tightrope after anatomically correct reduction is essential^26^. Nevertheless, it can be assumed that after reduction the placement of the positioning screw does not change the reduction result. This applies especially with regard to the weight-bearing situation and the functional follow-up treatment of the ankle joint.

Biomechanical and clinical studies have shown that a shortening of the fibula by more than 2 mm or rather its increased external rotation in the incisural notch by more than 5° results in a distinctive superior stress on the involved joint surfaces^8^. All reduction methods used in our study showed a comparably good result with regard to tibio-fibular adjustment, although the differences to the intact condition were significant. The deviations between the different methods were even smaller, so that it can be assumed that all methods are equally suitable for anatomically correct adjustment of the syndesmotic region and that marginal deviations can be tolerated, because their dimensions have no clinical relevance with regard to the development of premature degenerative alterations such as osteoarthritis.

The variance of the different reduction methods depending on the individual anatomical parameters can be explained by the equivalence of the techniques as well as by the small dimension of the measured distances and angles, which is limited by the pixel size of the radiological images.

A limitation of this study was that it did not include young, healthy patients because it was a specimen model with fresh-frozen lower legs from donors with an average age of 82.15 years. However, there is no evidence that degenerative changes influenced the data, as the specimens were screened for preexisting injury, surgery, osteoarthritis, and anatomic aberrations.

In addition, the injury conducted in the specimen model was limited to the syndesmotic ligaments in a non-weight bearing situation without an additional fibular fracture and therefore may not truly replicate the injury often found in the clinical setting. Nevertheless, the model simulates a complete lesion of the syndesmotic ligaments. In our opinion, a weight-bearing situation is not necessary for the comparison of the reduction methods, since on the one hand we use the images of the intact condition, which were also taken without weight bearing, and on the other hand the reduction instruments are only intended to support the final transfixation using a positioning screw or tightrope. A supplemental fibula fracture could certainly influence the forces of the ankle mortise. After accurate anatomic reduction and stable plate fixation, the impact is likely to be neglectable though. Therefore, we had no reason to believe that an additional fibula fracture would not be relevant for the results in this specimen model.

It can be assumed that the extremely high number of 38 lower-legs included in this specimen model generated reliable data.

Overall, the results show a satisfying reduction of the ankle mortise despite significant differences in terms of overcom- pression and the slight external rotation. There were no considerable differences between the reduction methods used. It can therefore be concluded that the ankle mortise can be reduced equally with any of the methods used but that the positioning and the contact pressure of each tool must be optimized. With regard to the hypothesis, the collinear reduction clamp turned out to be equivalent and a superior technique for reducing the syndesmotic region could not be identified.

## Methods

The study was approved by the Ethics Commission of the Medical Faculty of Heidelberg in accordance with the Declaration of Helsinki. The declarations of consent of the body donors are available at the Institute of Anatomy and Cell Biology of the University of Heidelberg.

The experimental cadaveric study was conducted on uninjured fresh frozen lower legs, which were disarticulated at the knee joint. The specimens were screened for prior injuries, surgeries, anatomical aberrations, and severe degenerative alterations. For the intervention, a metal-free, carbon fiber table was utilized to reduce artefacts. The three-dimensional imaging was obtained using a mobile cone beam CT (Arcadis Orbic, Siemens, Erlangen, Germany). The lower legs were positioned in an artefact-free acrylic glass plate at a 90° angle to achieve an exact 0°-neutral position of the ankle joint.

The induction of the syndesmotic injury and the surgical reduction were performed by an experienced specialist for trauma and orthopaedic surgery of a supraregional trauma center, while the radiological determination of the distances and angles as well as the statistical evaluation were carried out separately by an assistant physician for trauma and orthopaedic surgery.

### Study setup

**Figure 5:**
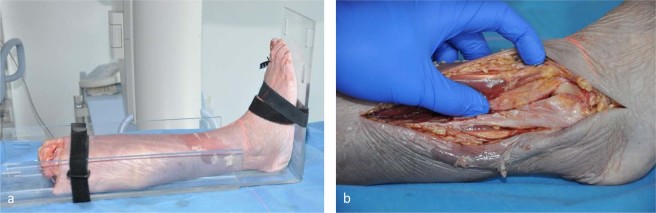
Photographs from the experimental test series: (**a**) Performing a 3D-Scan of the intact ankle in 0°-neutral position. (**b**) Dissection of the syndesmotic complex.

To obtain an image of the anatomically correct position of the intact ankle mortise the lower leg was placed in the acrylic plate on the carbon fiber table and a cone beam CT of the intact ankle in 0°-neutral position was performed (Fig. 5a). Through an anterolateral approach, the skin was incised laterally, and the distal fibula and the tibiofibular joint were exposed. The whole syndesmotic complex with the anterior inferior tibiofibular ligament (AiTFL), the interosseous membrane, the posterior inferior tibiofibular ligament (PiTFL), and the inferior transverse ligament (ITL) was consecutively dissected with all other periarticular ligaments being left intact (Fig. 5b).

**Figure 6:**
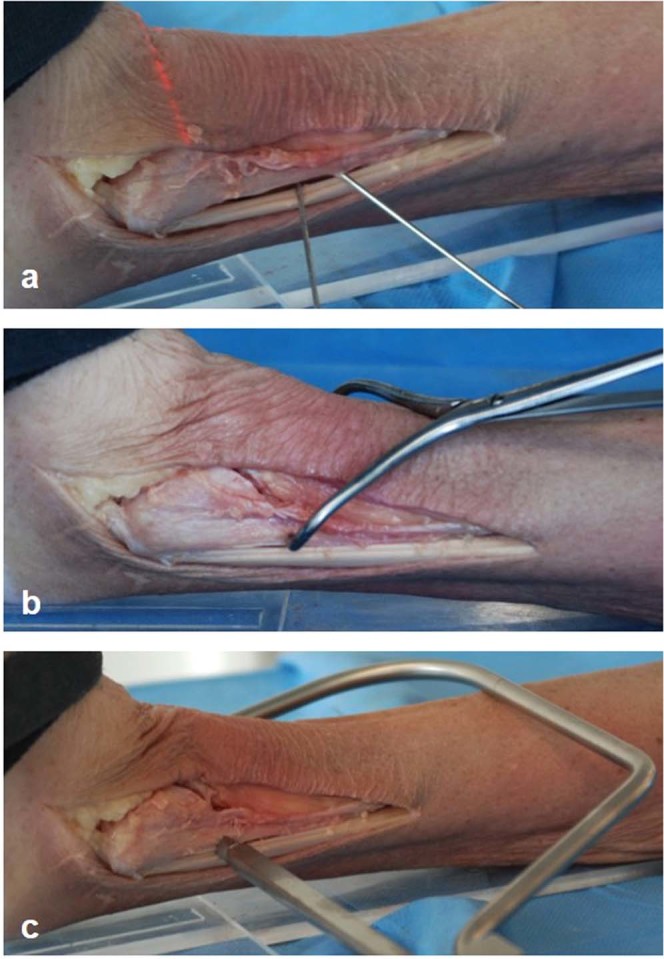
Photographs from the experimental test series: Reduction of the injured syndesmosis using (**a**) crossing K-wire transfixation, (**b**) conventional bone reduction forceps and (**c**) collinear reduction clamp.

**Figure 7:**
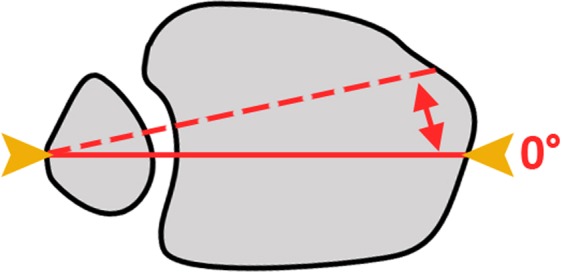
Sketch drawing: Axial view of the lower leg. Showing the clamp positioning (orange) in relation to the leg axis (red).

The displaced fibula then was reduced in the ankle mortise either manually combined with crossing K-wires transfixation (Fig. 6a), with conventional bone reduction forceps (Fig. 6b) or a collinear reduction clamp (Fig. 6c). The reduction clamps and the K-wires were placed in a 0° angle to the leg axis (Fig. 7). The clamps were positioned on the posterolateral ridge of the fibula approximately 20 mm proximal to the ankle joint line. The open reduction was performed until a satisfying clinical result was palpable and visible.

A cone beam CT scan was performed after every reduction method. In total five scans of each extremity were evaluated.

### Radiological analysis

**Figure 8:**
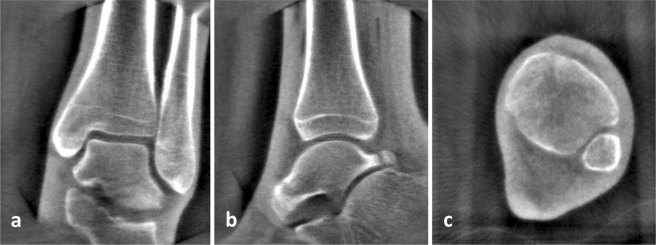
Screenshots of multiplanar reconstructions: Ankle in (**a**) coronal, (**b**) sagittal and (**c**) axial view.

The images were analyzed with the software “syngo” (Siemens, Medical Healthcare, Erlangen, Germany). Distances and angles of the ankle mortise were assessed. According to a standard CT the sagittal and coronal planes were reconstructed (Fig. 8).

**Figure 9:**
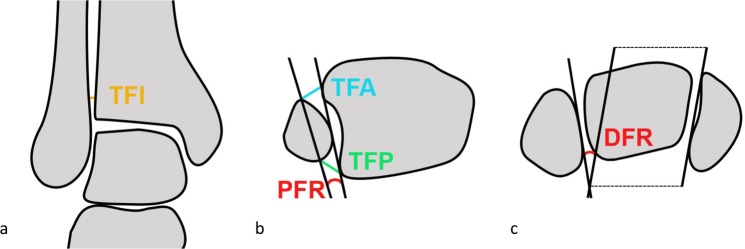
Sketch drawings: (**a**) Determination of the tibiofibular distance in the incisural notch (TFI, orange) in the coronal plane. (**b**) Determination of the anterior tibiofibular distance (TFA, light blue), the posterior tibiofibular distance (TFP, green) and the proximal angle of the fibular rotation (PFR, red) in the axial plane. (**c**) Determination of the distal angle of the fibular rotation (DFR, red) in the axial plane.

In the coronal plane, the tibiofibular distance in the incisural notch (distance TFI – 10 mm proximal to the tibial joint line) was identified (Fig. 9a). In the axial plane, 10 mm proximal to the tibial articular surface the anterior tibiofibular distance (distance TFA - between the anterior edge of tibia and fibula) and the posterior tibiofibular distance (distance TFP -between the posterior edge of tibia and fibula), as well as the proximal angle of the fibular rotation (angle PFR) (Fig. 9b) were measured. The distal fibular rotation (angle DFR) was also assessed 5 mm distal to the talar articular surface (Fig. 9c).

### Statistical analysis

SPSS (IBM Corporation, Armonk, New York, USA) was used for the statistical analysis. Means and standard deviations of all measurements were obtained. A t-test for paired samples was applied to identify statistical significance. The significance threshold was set at *p* ≤ 0.05. Since the data sets are approximately normally distributed, further statistical investigations are not necessary to compare the reduction methods in terms of reduction quality.

### Accession codes

The study was approved by the ethics committee of the Medical Faculty of the University of Heidelberg. The application was submitted on 13.01.2014 and was accepted on 17.02.2014 with the registration number S-013/2014.
